# Supplementary material for: Circulating ACE2-expressing extracellular vesicles block broad strains of SARS-CoV-2
Source: Nat Commun. 2022 Jan 20;13:405. doi: 10.1038/s41467-021-27893-2 (PMC8776790; doi:10.1038/s41467-021-27893-2)
Supplement: Supplementary file 1 — Supplementary Information [file 41467_2021_27893_MOESM1_ESM.pdf]

Circulating ACE2-expressing Extracellular Vesicles as A Decoy Therapy to Block Broad Strains of SARS-CoV-2

Supplementary Tables 1-3 and Figures 1-6

Lamiaa El-Shennawy[1*], Andrew D. Hoffmann[1*], Nurmaa K. Dashzeveg[1*], Kathleen M. McAndrews[2*], Paul J. Mehl[3], Daphne Cornish[1], Zihao Yu[1], Valerie L. Tokars[1], Vlad Nicolaescu[4], Anastasia Tomatsidou[4], Chengsheng Mao[5], Christopher J. Felicelli[6], Chia-Feng Tsai[7], Carolina Ostiguin[3], Yuzhi Jia[1], Lin Li[8], Kevin Furlong[6], Jan Wysocki[9],Xin Luo[2], Carolina F. Ruivo[2], Daniel Batlle[9], Thomas J. Hope[10], Yang Shen[11], Young Kwang Chae[12], Hui Zhang[8], Valerie S LeBleu[1,2], Tujin Shi[7], Suchitra Swaminathan[3,13], Yuan Luo[5], Dominique Missiakas[4], Glenn C. Randall[4], Alexis R Demonbreun[1], Michael G Ison[14,15], Raghu Kalluri[2,16, 17#], Deyu Fang[3,6#], Huiping Liu[1,3,12#]

[1]Department of Pharmacology, Northwestern University Feinberg School of Medicine, Chicago, IL, USA 60611. [2] Department of Cancer Biology, The University of Texas MD Anderson Cancer Center, Houston, TX, USA 77030, [3]Robert H. Lurie Comprehensive Cancer Center, Northwestern University Feinberg School of Medicine, Chicago, IL, USA 60611. [4]The University of Chicago Howard T. Ricketts Laboratory and Department of Microbiology, Chicago, IL 60637. [5]Division of Health and Biomedical Informatics, Department of Preventive Medicine, [6]Department of Pathology, Northwestern University Feinberg School of Medicine, Chicago, IL, USA 60611. [7]Biological Sciences Division, Pacific Northwest National Laboratory, Richland, Washington 99354, USA. [8]Division of Biostatistics, Department of Preventive Medicine, [9]Division of Nephrology and Hypertension, Department of Medicine, [10]Department of Cell and Developmental Biology, Northwestern University Feinberg School of Medicine, Chicago, IL, USA 60611. [11]Department of Electrical and Computer Engineering, TEES-AgriLife Center for Bioinformatics and Genomic Systems Engineering, Texas A&M University, College Station, TX 77843. [12]Division of Hematology and Oncology, [13]Division of Rheumatology, Department of Medicine, [14]Division of Infectious Disease, Department of Medicine and Division of Organ Transplantation, [15]Department of Surgery, Northwestern University Feinberg School of Medicine, Chicago, IL, USA 60611. [16]Department of Bioengineering, Rice University, Houston, TX, USA 77005. [17]Department of Molecular and Cellular Biology, Baylor College of Medicine, Houston, TX, USA 77030.
[*]These authors contributed equally

[#]These authors jointly supervised this work. Huiping Liu, MD, PhD, Northwestern University, 303 E Superior St, Chicago, IL 60611. Email: huiping.liu@northwestern.edu. Deyu Fang, MD, PhD, Northwestern University, 303 E Chicago St, Chicago, IL 60611. Email: fangd@northwestern.edu. Raghu Kalluri, MD, PhD, The University of Texas MD Anderson Cancer Center, 1881 East Rd, Houston, TX 77054. Email: RKalluri@mdanderson.org.

Supplementary Table 1. Antibodies conditions for immunoblotting.

Antibody	Vendor	Catalog number	Dilution	Figure
ACE2	Cell Signaling	4355S	1:1,000	Suppl. Fig. 2c, e
CD81 (B-11)	Santa Cruz Biotechnology	sc166029	1:20,000	Suppl. Fig. 2e
His-tag	Cell Signaling	2365S	1:1,000	Suppl. Fig. 2c, e
HSP90 (C45G5)	Cell Signaling	4877S	1:1,000	Suppl. Fig. 2e
Syntenin (EPR8102)	Abcam	ab133267	1:20,000	Suppl. Fig. 2c
Rabbit IgG HRP-Conjugated	Abcam	ab16284	1:2,000	Suppl. Fig. 2c, e
Rabbit IgG HRP-Conjugated	Cell Signaling	7074S	1:5,000	Suppl. Fig. 2c
Mouse IgG HRP-Conjugated	R&D Systems	HAF007	1:5,000	Suppl. Fig. 2c, e
ACE2	R&D Systems	AF933	1:1,000	Fig. 1f, Suppl. Fig. 1e Suppl. Fig. 3e Suppl. Fig 6e
CD81	GeneTex	GTX101766	1:1,000	Fig. 1f
CD63	Proteintech	25682-1-AP	1:1,000	Fig. 1f
GRP94 (1H10B7)	Proteintech	60012-1-Ig	1:1,000	Fig. 1f
TSG101	Proteintech	14497-1-AP	1:1,000	Fig. 1f, Suppl. Fig. 1e Suppl. Fig. 6e
Goat IgG HRP-conjugated	Thermo Fisher Scientific	31410	1:2000	Fig. 1f, Suppl. Fig. 1e Suppl. Fig. 3e Suppl. Fig 6e
Rabbit IgG HRP-Conjugated	Promega	W401B	1:10,000	Fig. 1f, Suppl. Fig. 1e Suppl. Fig. 6e
Mouse IgG HRP-Conjugated	Promega	W402B	1:10,000	Fig. 1f

Supplementary Table 2: Depletion conditions with RBD conjugation and beads

EV samples	Volume of EV samples (µL)	Total RBD (µg)	Volume of RBD-beads (µL) for RBD conjugation
CSB-012	125	2	50
CSB-024	125	2	50
CBB-008#	20	6	150*
CBB-009#	20	6	150*
CBB-0012	20	6	150*
CBB-0013#	20	6	150*
$ACE2^{+}$EV (5 µg)	20	6	150*

*Beads were spun with buffer removed before the EV sample was added for depletion.
#Plasma EVs were diluted 1:2 before depletion.

Supplementary Table 3: Histopathological Grading

Scoring	Acute Inflammation	Chronic Inflammation	Hyaline Membranes	Hemorrhage	Necrosis
0	None	0%	0%	0%	0%
1	Scattered in interstitium	1-5%	1-5%	1-5%	1-5%
2	Extravasated in airspaces	6-25%	6-25%	6-25%	6-25%
3	Small aggregates in vessels	26-50%	26-50%	26-50%	26-50%
4	Large aggregates in vessels and airways	51-75%	51-75%	51-75%	51-75%
5	Diffuse involvement	76-100	76-100%	76-100%	76-100%

Supplementary Fig. 1: MFV analysis of evACE2 in COVID-19 plasma and SARS-CoV-2 infected cells

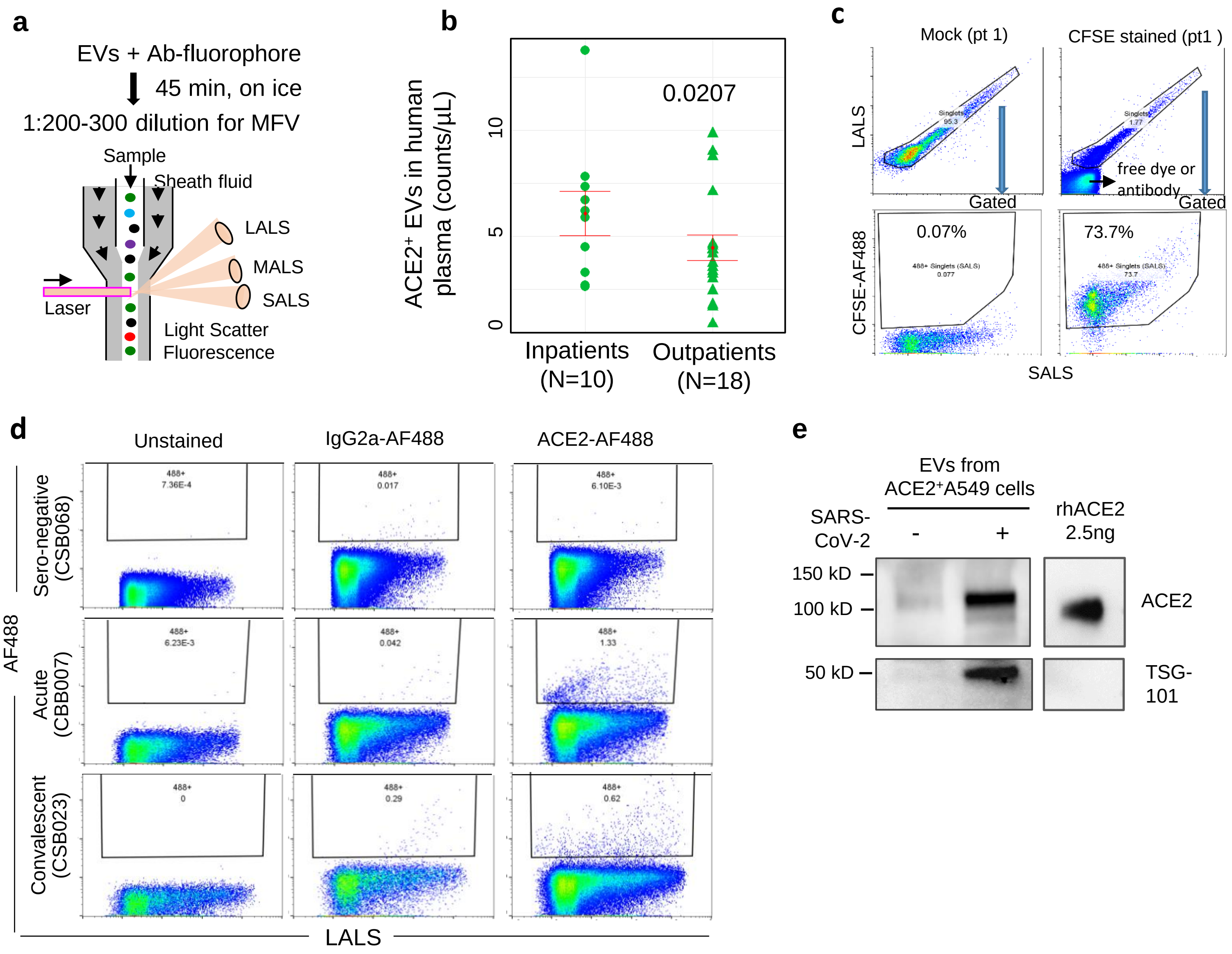

Supplementary Fig 1. MFV analysis of evACE2 in COVID-19 plasma and SARS-CoV-2 infected cells.

a. EV staining protocol for Apogee-based microflow vesiclometry (MFV) on fluorescent and light scatter panels, SALS, MALS, and LALS.

b. MFV of $ACE2^+$ EV counts in human COVID-19 convalescent plasma of hospitalized and outpatients. One-tail *t* test, * p=0.0207. Data are presented as mean values +/- SEM

c. Light scatter channels (LALS and SALS) of patient specimen with or without CFSE staining showing the separate subset of small participle of free dye which can be used to gate certain antibody conjugates if not removed by centrifugation. In our ACE2 antibody and CD63 antibody staining, the antibody dye subsets were not present.

d. MFV plots of unstained, IgG2a-AF488, and ACE2-AF488 stained patient plasma sample representatives of sero-negative control CSB-068, acute COVID-19 CBB-007, and convalescent CSB-023). The final counts are normalized after deducting the IgG isotype control noise for each patient plasma sample.

e. Immunoblots of ACE2 and TSG101 of EVs ultracentrifuged (100,000 g x 8 h at 4°C after removing cell debris) from the culture supernatant of ACE2-overexpressing A549 cells with or without SARS-CoV-2 infection (72 h).). Equal volumes of supernatant were processed/loaded, and RIPA buffer was used to lyse isolated EVs (n=2 biological replicates).

Supplementary Fig. 2: Detection of evACE2 in human plasma and engineered cell culture

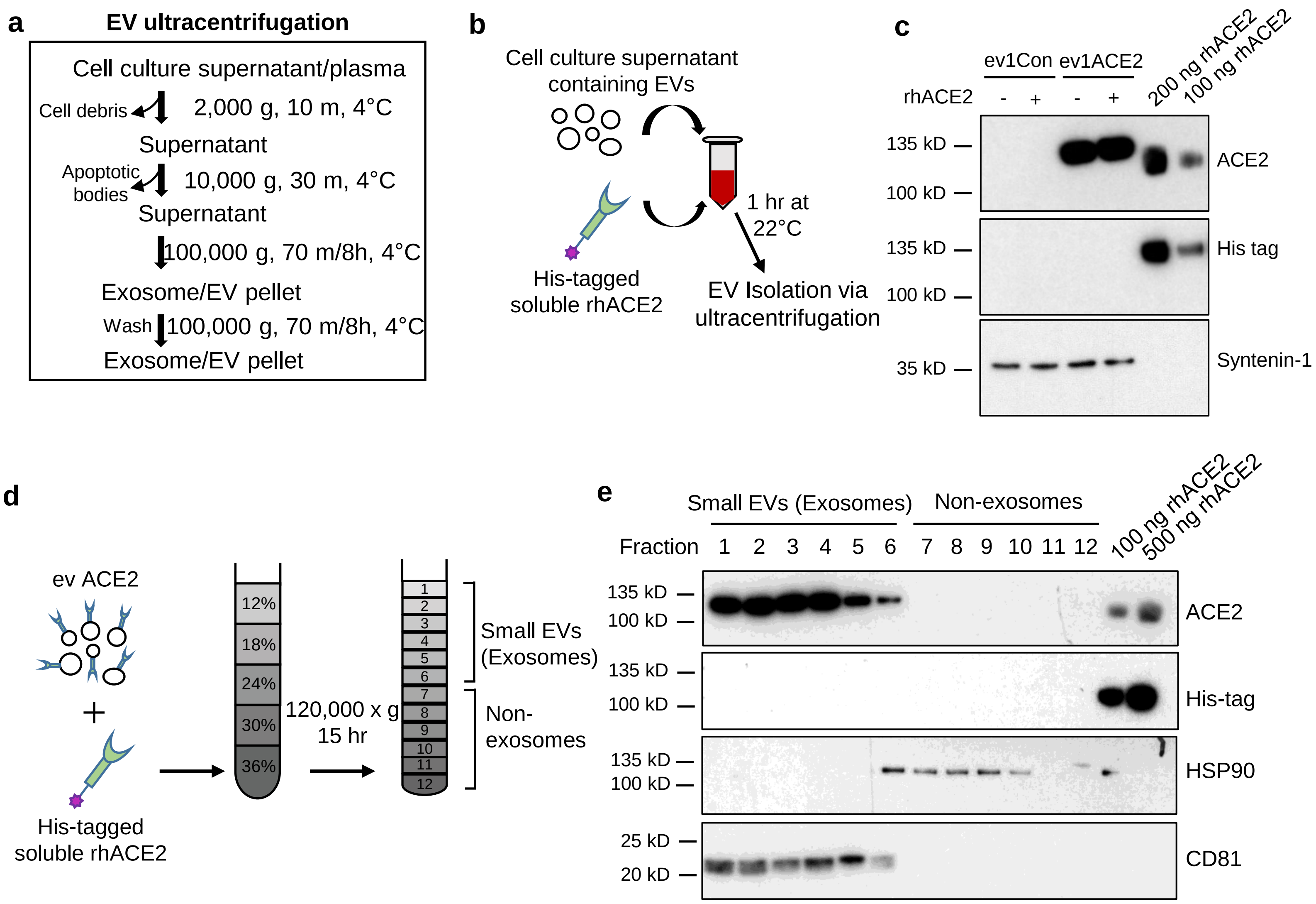

Supplementary Fig 2. Detection of evACE2 in human plasma and engineered cell culture.

a. Exosome-enriched EV isolation protocol by ultracentrifugation.

b. Schematic depiction of experiments evaluating soluble vs. vesicular ACE2.

c. Immunoblots for ACE2, His-tag, and syntenin-1 in EVs isolations from the supernatant of HEK (con EV) and HEK ACE2 (ACE2 EV) with or without spiking of rhACE2 (2 µg). 100 and 200 ng of rhACE2 were directly loaded into the gels as positive controls for soluble ACE2. EVs were measured in PBS via Nanodrop and then lysed in urea buffer. N=2 independent experiments.

d. Schematic depiction of Optiprep density gradient fractionation of ACE2 EVs isolated by ultracentrifugation.

e. Immunoblots for ACE2, His-tag, HSP90, and CD81 of density gradient fractionated HEK ACE2 EVs. EVs were measured in PBS via Nanodrop and then lysed in urea buffer. N=2 independent experiments.

Supplementary Fig. 3: Analyses of evACE2 via Cryo-EM, MFV, ELISA and immuno-blotting

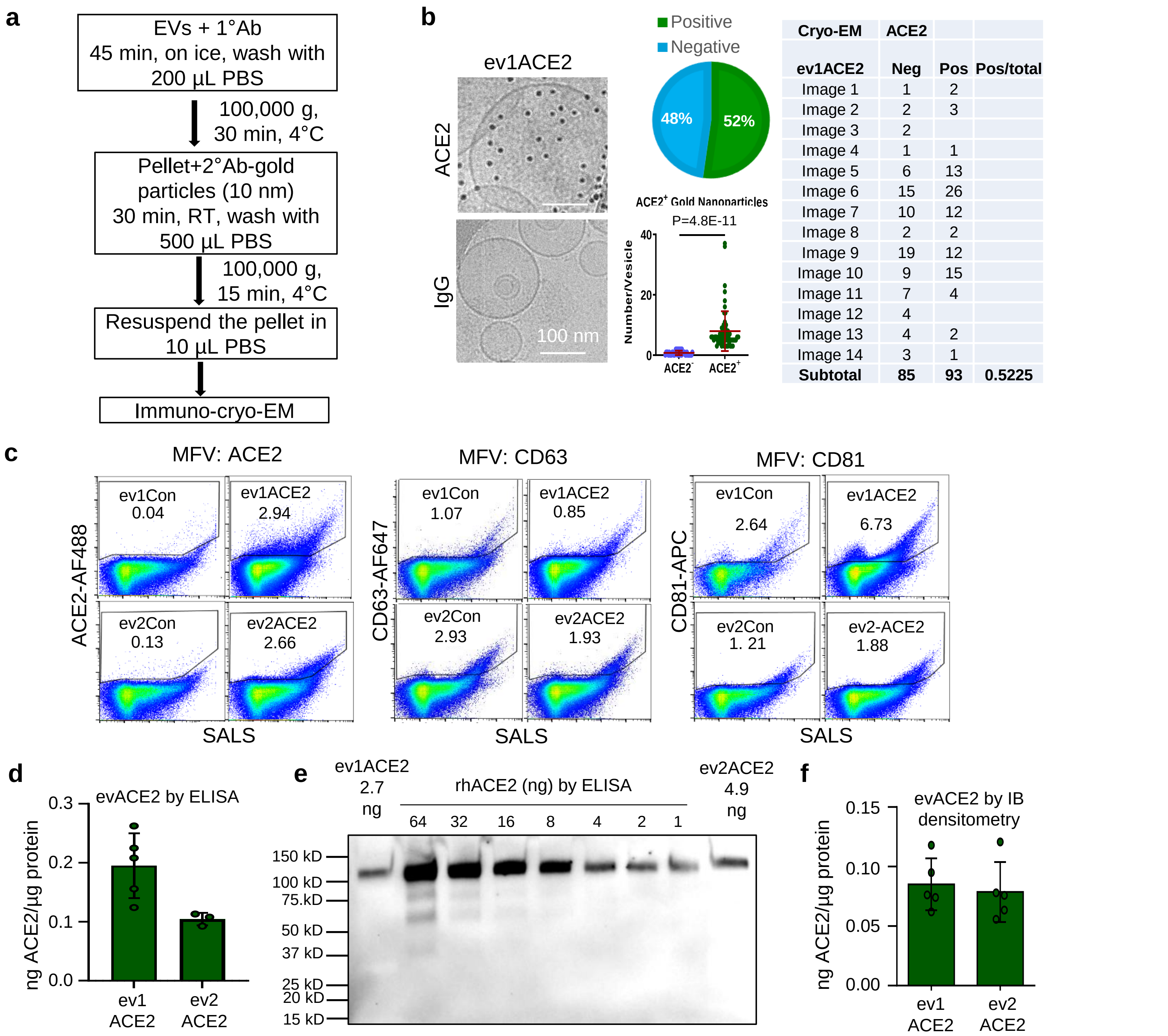

Cryo-EM	ACE2		
ev1ACE2	Neg	Pos	Pos/total
Image 1	1	2	
Image 2	2	3	
Image 3	2		
Image 4	1	1	
Image 5	6	13	
Image 6	15	26	
Image 7	10	12	
Image 8	2	2	
Image 9	19	12	
Image 10	9	15	
Image 11	7	4	
Image 12	4		
Image 13	4	2	
Image 14	3	1	
**Subtotal**	**85**	**93**	**0.5225**

Supplementary Fig. 3. Analyses of evACE2 via Cryo-EM, MFV, ELISA and immuno-blotting

a. Immuno-cyro-EM staining protocol with primary antibody and secondary antibody (conjugated with gold particles.

b. Cryo-EM image of $ACE2^+$ EVs, a pie graph (table), and plot of counted HEK-$ACE2^+$ (n=64 biologically independent vesicles) and $ACE2^-$ (n=45 biologically independent vesicles) EVs. Two-tail *t* test (****p=4.8E-11). Data are presented as mean values +/- SD.

c. MFV profiles of exosomal CD63, CD81 and ACE2 expression in four cell line-derived EVs (HEK and HeLa with or without ACE2 expression). The final counts are normalized after deducting the IgG isotype control noise for each sample. N=2 independent experiments with n=3 technical replicates each.

d. ELISA-based detection of ACE2 in ev1ACE2 (HEK) and ev2ACE2 (HeLa) (n=5 and 3 biologically independent samples, respectively). EVs measured by Nanodrop in PBS. Data are presented as mean values +/- SD.

e. Immunoblots of ACE2 in purified ev1ACE2 (HEK) and ev2ACE2 (HeLa) in comparison to a serial dilution of rhACE2 (1-64 ng as measured by ELISA). EV proteins were measured in PBS by Nanodrop (27.3 and 87.5 μg for ev1 and ev2, respectively) and then lysed using RIPA buffer (N=3 independent experiments)..

f. The amount of ACE2 in EVs (ng ACE2/μg EV protein) is determined by densitometry of rhACE2 standards, within the range as measured by ELISA (Supplementary Fig 3d) (n=5 biologically independent samples). Data are presented as mean values +/- SD.

Supplementary Fig. 4: evACE2 blocks RBD binding to ACE2+ HEK cells

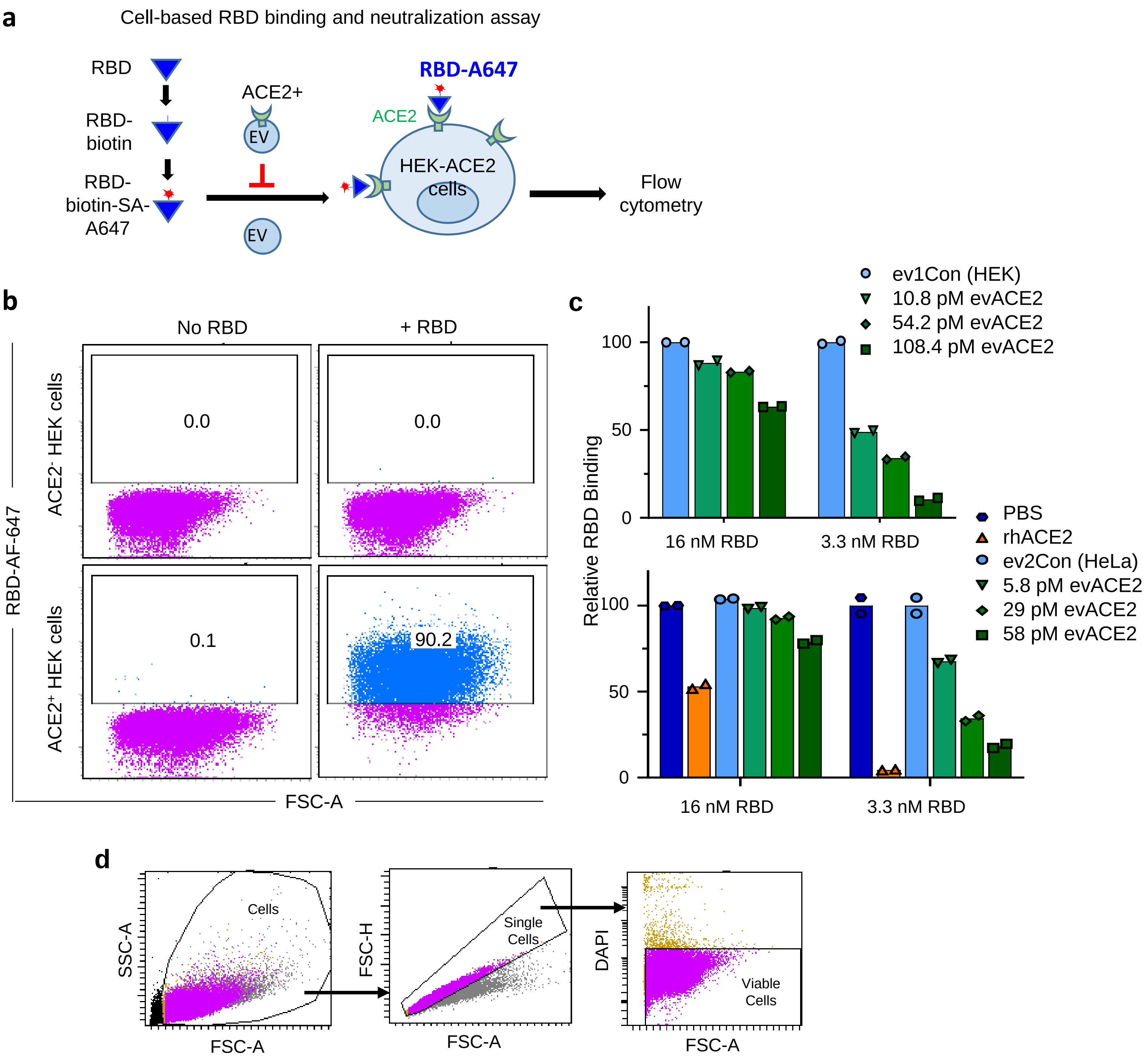

Supplementary Fig. 4: evACE2 blocks RBD binding to ACE2+ HEK cells.

a. Schematic of flow cytometry-based RBD binding to human host cells (ACE2$^+$ HEK) for neutralization effect analysis

b. Flow plots of ACE2$^+$ and ACE2$^-$ HEK cells in the absence and presence of AF647-conjugated RBD binding with minimal binding to a mock control of the RBD probe.

c. Histogram bars of quantified RBD-neutralization by evACE2 (ev1 from HEK and ev2 from HeLa) in a dose dependent manner in comparison to ACE2$^-$ parental cell derived control EVs (ev1Con and ev2Con from HEK and HeLa, respectively(n=2 technical replicates). PBS (dark blue), evCON (orange), rhACE2 (orange) and evACE2 (light green , green and dark green).

d. Gating Strategy: DAPI negative viable single cells based on FSC, SSC and FSH are gated for percentage and mean fluorescence intensity (MFI).

Supplementary Fig. 5: evACE2 blocks pseudotyped and authentic SARS-CoV-2 infections to cells

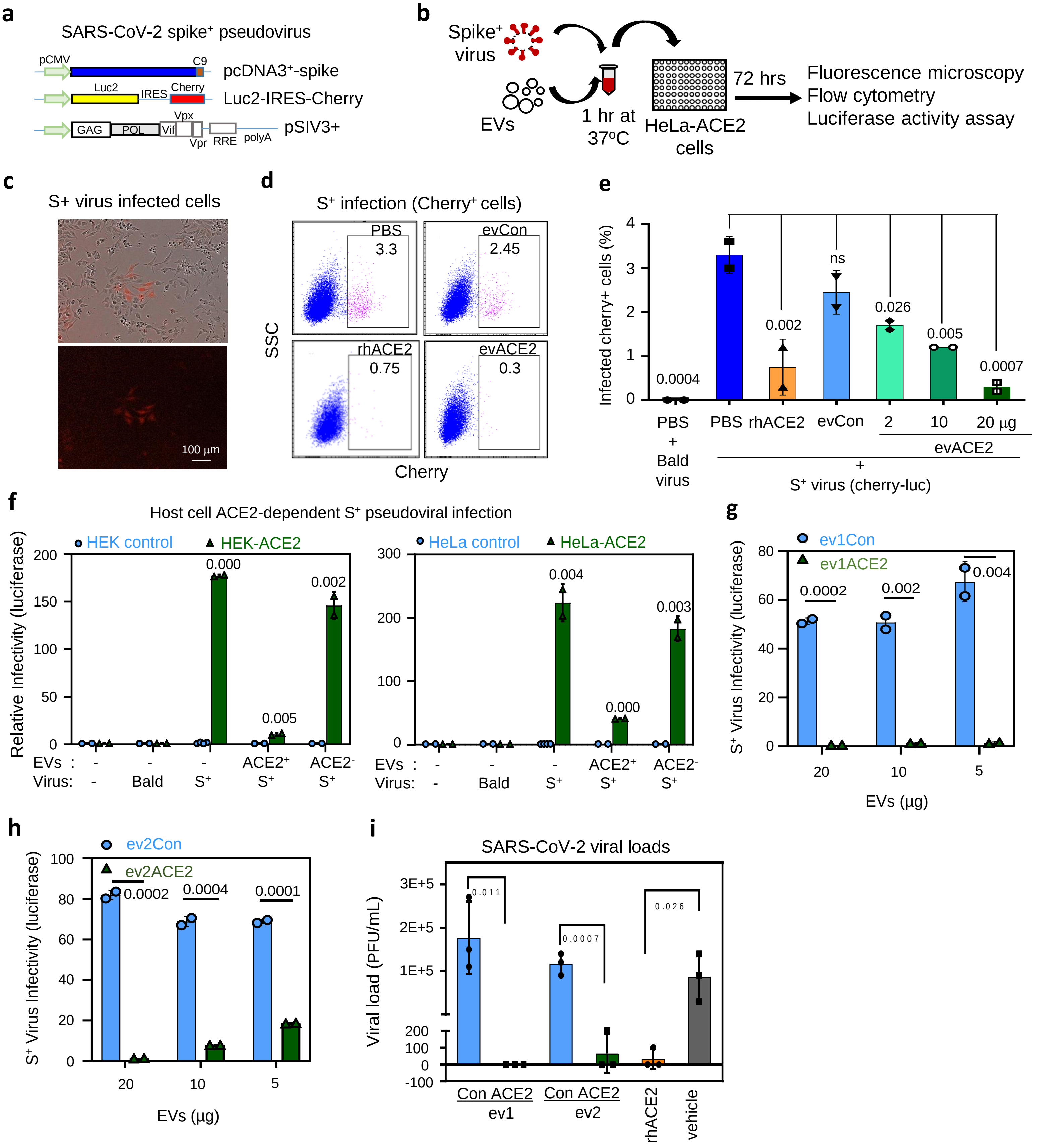

Supplementary Fig 5: ACE2$^+$ EVs block pseudotyped and authentic SARS-CoV-2 infections to host cells.

a. Schematic of the three vectors for SARS-CoV-2 S$^+$ pseudovirus production.
b. Pseudovirus infection with ACE2$^+$ HeLa cells and subsequent infectivity analyses, including Cherry positive cells under fluorescent microscopy, MFV, and luciferase activity assays.
c. Fluorescent images of Cherry$^+$ red cells as pseudovirus-infected cells (N=5 independent experiments).
d-e. Flow plots (**d**) and bar graph **(e)** of pseudovirus-infected HeLa-ACE2 cells, detected with Cherry reporter expression which was inhibited by rhACE2 (orange) and ACE2$^+$ Evs (light green, green and dark green), but not ACE2$^-$ Evs (light blue). One-way ANOVA followed by Tukey's multiple comparisons (*p=0.026, **p=0.005, **p=0.002, ***p=0.0007, ***p=0.0004 and ns=non-significant compared to PBS+Spike). N=2 experiments with two technical replicates each. Data are presented as mean values +/- SD.
f. Luciferase-based viral infectivity between ACE2$^-$ control (light blue) and ACE2$^+$ HEK (green) cells (left panel) and between ACE2$^-$ control and ACE2$^+$ HeLa cells (right panel), 72 hr after incubation with bald (S$^-$) or S$^+$ pseudotyped viruses in the absence and presence of ACE2$^+$ or ACE2$^-$ EVs (****p<0.0001). One-tail *t* test, **p=0.002, **p=0.003, **p=0.004, **p=0.005 and ****p=0.000 (<0.0001) compared to respective no virus. N=2 experiments with two technical replicates each.
g-h. Luciferase-based SARS-COV-2 S$^+$ pseudotype infectivity of HeLa-ACE2 cells in the presence of ACE2$^-$ (light blue) and ACE2$^+$ (green) EV2s from both HEK (g) and HeLa (h) cells (N=2 experiments with two technical replicates each). One-tail *t* test, **p=0.004, **p=0.002, ***p=0.0004, ***p=0.0002 and ***p=0.0001 compared to respective ACE2- EVs).
i. SARS-CoV-2 viral loads of Vero-6 host cells after viral infections in the presence of 10 µg EV1/EV2 protein measured via Nanodrop (control (light blue) and ACE2$^+$ (green), ~ 1ng as determined by ELISA), rhACE2 (orange) (128 ng as determined by ELISA), and vehicle (grey). n=3 biological replicates. One-tail t test, *p=0.026, *p=0.011 and ***p=0.0007.

Supplementary Fig 6. Patient plasma analysis and mouse biodistribution of therapeutic evACE2

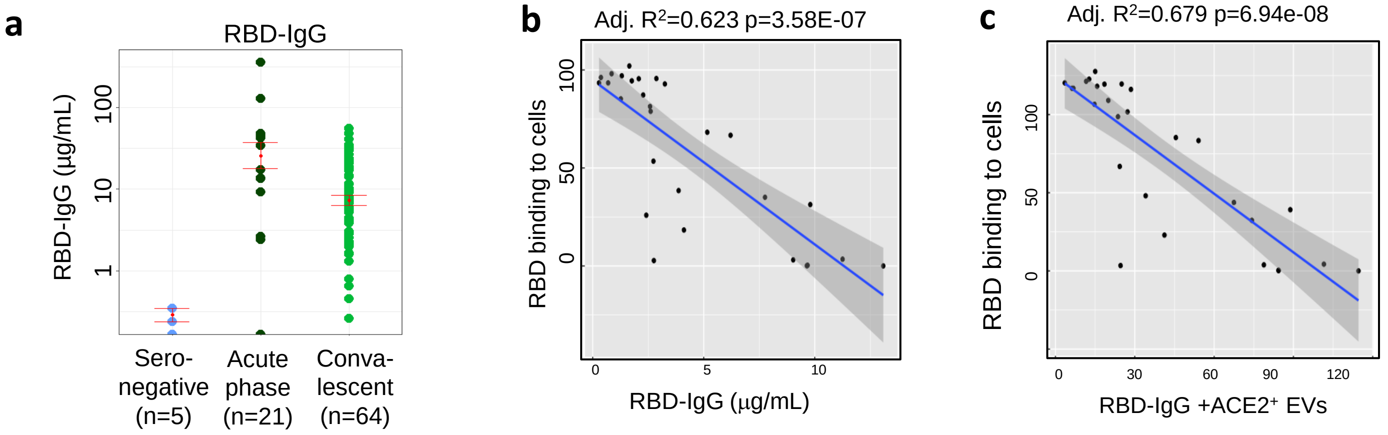

d

Patient plasma	RBD [IgG] µg/ml
Seronegative control	0.00
CBB_005 T1 acute	0.00
CBB_007 T1 acute	0.00
CBB_008 T1 acute	46.76
CBB_009 T1 acute	45.80
CBB_012 T1 acute	0.00
CBB_013 T1 acute	128.62
CSB-012 -convalescent	6.21
CSB-024 -convalescent	2. 61

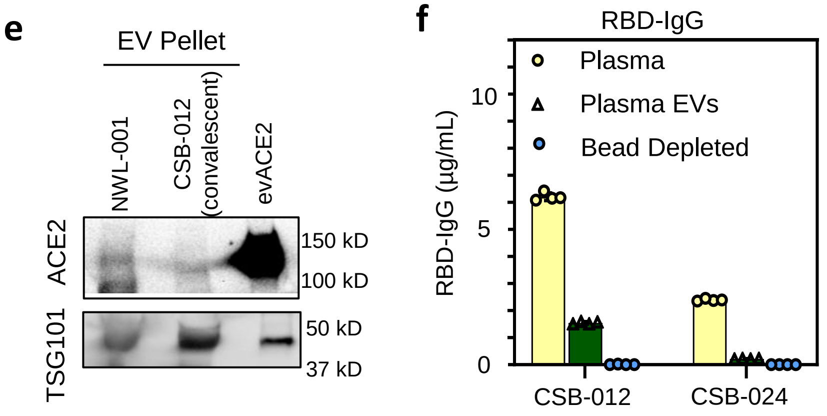

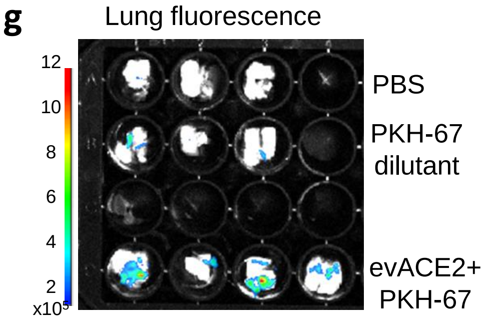

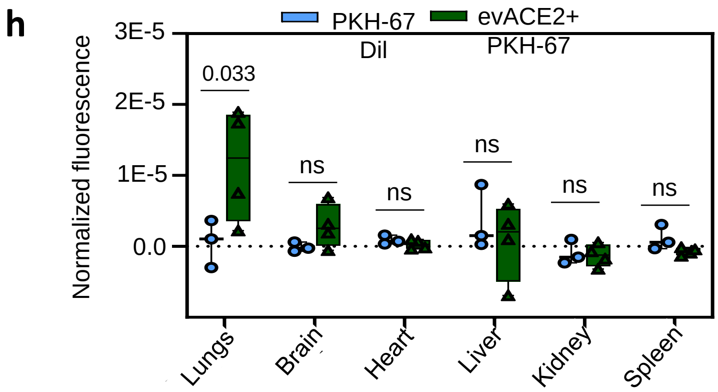

Supplementary Fig 6. Patient plasma analysis and mouse biodistribution of therapeutic evACE2.

a- RBD-IgG levels detected in human plasma of sero-negative (light blue), acute phase (dark green), and convalescent COVID-19 (light green). One-tail *t* test. Data are presented as mean values +/- SEM .

b-c. Negative correlation between COVID-19 plasma RBD-IgG levels and the RBD-host cell binding. Adjusted R square 0.623 with P=3.58E-07 (b). Negative association of RBD binding to host cells with the integrated RBD-IgG and ACE2$^+$ EV levels in the convalescent COVID-19 patient plasma (c). N=30 and shaded areas represent error bands. One-way ANOVA was performed to compare group means (F=0.01, p-value>0.9). Poisson regression was fitted to estimate the associations between normalized percentage of RBD binding to cells and independent predictors of interest.

d. RBD-IgG levels in plasma samples of sero-negative control and acute and convalescent patients.

e. Immunoblots of plasma EV pellets for ACE2 and TSG-101, isolated from NWL-001 (lung cancer patient), and a COVID-19 convalescent patient CSB-012 (N=1 experiment).

f: Minimal or residual RBD-IgG in plasma EV pellet prior to (green) and after RBD (light blue) bead depletion compared to crude plasma (yellow) of convalescent COVID-19 patients (CSB-012 and -024) (n=4).

g. Fluorescence images of dissected lungs of B6 mice at 24 h post intranasal delivery of PBS, PKH-67 dilutant buffer control without EVs, and PKH-67-labeled evACE2 (130 µg/mouse).

h. Levels of fluorescent signals (evACE2 biodistribution) to the various organs, with the most majority to the lungs and not significant distribution to brain, heart, liver, kidney and spleen (N=3 and 4 for PKH-67 Dil (light blue) and evACE2+ PKH-67 (green), respectively). Non- parametric one-tail *t* test (*p=0.033 and ns=non-significant). Whiskers represent minimum to maximum showing all points, the box extends from 25th to 75th percentiles, and the line in the middle of the box is plotted at the median.
