# Supplementary material for: Circulating ACE2-expressing extracellular vesicles block broad strains of SARS-CoV-2
Source: Nat Commun. 2022 Jan 20;13:405. doi: 10.1038/s41467-021-27893-2 (PMC8776790; doi:10.1038/s41467-021-27893-2)
Supplement: Supplementary file 3 — Description of Additional Supplementary Files [file 41467_2021_27893_MOESM3_ESM.pdf]

Description of Additional Supplementary Files

File Name: Supplementary Data 1
Description: Mass spectrometry data of the peptides pulled down by RBD-beads
