# Supplementary material for: Circulating ACE2-expressing extracellular vesicles block broad strains of SARS-CoV-2
Source: Nat Commun. 2022 Jan 20;13:405. doi: 10.1038/s41467-021-27893-2 (PMC8776790; doi:10.1038/s41467-021-27893-2)
Supplement: Supplementary file 5 — Reporting Summary [file 41467_2021_27893_MOESM5_ESM.pdf]

nature research

Corresponding author(s): Huiping Liu

Last updated by author(s): December 28, 2021

Reporting Summary

Nature Research wishes to improve the reproducibility of the work that we publish. This form provides structure for consistency and transparency in reporting. For further information on Nature Research policies, see our Editorial Policies and the Editorial Policy Checklist.

Statistics

For all statistical analyses, confirm that the following items are present in the figure legend, table legend, main text, or Methods section.

n/a	Confirmed	
[ ]	[x]	The exact sample size (*n*) for each experimental group/condition, given as a discrete number and unit of measurement
[ ]	[x]	A statement on whether measurements were taken from distinct samples or whether the same sample was measured repeatedly
[ ]	[x]	The statistical test(s) used AND whether they are one- or two-sided<br>*Only common tests should be described solely by name; describe more complex techniques in the Methods section.*
[ ]	[x]	A description of all covariates tested
[x]	[ ]	A description of any assumptions or corrections, such as tests of normality and adjustment for multiple comparisons
[ ]	[x]	A full description of the statistical parameters including central tendency (e.g. means) or other basic estimates (e.g. regression coefficient) AND variation (e.g. standard deviation) or associated estimates of uncertainty (e.g. confidence intervals)
[ ]	[x]	For null hypothesis testing, the test statistic (e.g. *F*, *t*, *r*) with confidence intervals, effect sizes, degrees of freedom and *P* value noted<br>*Give P values as exact values whenever suitable.*
[x]	[ ]	For Bayesian analysis, information on the choice of priors and Markov chain Monte Carlo settings
[x]	[ ]	For hierarchical and complex designs, identification of the appropriate level for tests and full reporting of outcomes
[x]	[ ]	Estimates of effect sizes (e.g. Cohen's *d*, Pearson's *r*), indicating how they were calculated

Our web collection on statistics for biologists contains articles on many of the points above.

Software and code

Policy information about availability of computer code

Data collection

1. Cells were diluted in PBS and filtered, prior to analysis on a BD FACSAria SORP or BD-LSR II flow cytometer (BD Biosciences)
2. For pseudo virus experiments cultured cells were incubated and monitored by the IncuCyte live cell imaging system (Essen BioScience), and images were acquired every 2 h.
3. EVs were diluted in PBS prior to analysis on Apogee A50 Micro Flow Cytometer (MFC) (Apogee Flow Systems, Hertfordshire, UK) (http://www.apogeeflow.com/products.php) or NanoSight NS3000.
4. For Immuno-cryo-EM, EVs were imaged using a JEOL 3200FS electron microscope equipped with an omega energy filter operated at 200 kV with a K3 direct electron detector (Ametek) using the minimal dose system.
5. For immunoblots, BioRad ChemiDoc imaging System was used to collect data.
6. Optical density and luciferase activity were measured using BioTek Synergy HT or infinite 200Pro.
7. Ex Vivo imaging was done using LAGO from Spectral Imaging Instruments .

Data analysis	Please refer to the method sections for details.<br>1. Flow cytometry and microflow cytometry data have been analyzed by BD FACSDiva softwares v8.0.2 or v8.0.3 or Flow Jo v10.6.2.<br>2. Ex vivo imaging data were analysed using Aura imaging software.<br>3. IC50 was determined using GraphPad Prism 9.0.2.<br>4. Image Lab 6.1 was used for densitometry quantification .<br>5. The R program codes used in the data analysis are available in https://github.com/adhoffma/Liu_Lab/tree/main/El-Shennawy%20et%20al%202021. R 4.0.2 was used.<br>6. The cellular components annotation was analyzed by DAVID v6.8.

For manuscripts utilizing custom algorithms or software that are central to the research but not yet described in published literature, software must be made available to editors and reviewers. We strongly encourage code deposition in a community repository (e.g. GitHub). See the Nature Research guidelines for submitting code & software for further information.

Data

Policy information about availability of data

All manuscripts must include a data availability statement. This statement should provide the following information, where applicable:

- Accession codes, unique identifiers, or web links for publicly available datasets
- A list of figures that have associated raw data
- A description of any restrictions on data availability

Mass spectroscopy raw data sets have been deposited in the Japan ProteOmeSTandard Repository [Nucleic Acids Res. 2017, 45 (D1), D1107−D1111] (https://repository.jpostdb.org/). The accession numbers are PXD029662 for ProteomeXchange [Nat. Biotechnol. 2014, 32 (3), 223−6] and JPST001379 for jPOST. Data that support findings of this study have been included in the Source data file.

Field-specific reporting

Please select the one below that is the best fit for your research. If you are not sure, read the appropriate sections before making your selection.

- [x] Life sciences
- [ ] Behavioural & social sciences
- [ ] Ecological, evolutionary & environmental sciences

For a reference copy of the document with all sections, see nature.com/documents/nr-reporting-summary-flat.pdf

Life sciences study design

All studies must disclose on these points even when the disclosure is negative.

Sample size	For in vitro experiments, sample sizes was determined based on our pilot experiments.<br>For the animal study, we determined the sample size using Bonferroni correction for multiplicity to achieve 80% power using the two-sample t-test.
Data exclusions	no data exclusions.
Replication	Almost all experiments were replicated at least two times. All attempts at replication were successful.
Randomization	Mouse groups were randomized. All other experiments were conducted with randomly allocated samples and groups.
Blinding	The data collection and analyses of mouse experiment were blinded.

Reporting for specific materials, systems and methods

We require information from authors about some types of materials, experimental systems and methods used in many studies. Here, indicate whether each material, system or method listed is relevant to your study. If you are not sure if a list item applies to your research, read the appropriate section before selecting a response.

Materials & experimental systems

n/a	Involved in the study	
[ ]	[x]	Antibodies
[ ]	[x]	Eukaryotic cell lines
[x]	[ ]	Palaeontology and archaeology
[ ]	[x]	Animals and other organisms
[ ]	[x]	Human research participants
[ ]	[x]	Clinical data
[x]	[ ]	Dual use research of concern

Methods

n/a	Involved in the study	
[x]	[ ]	ChIP-seq
[ ]	[x]	Flow cytometry
[x]	[ ]	MRI-based neuroimaging

Antibodies

Antibodies used	For immuno blotting: ACE2 (Cell signaling, 4355S, 1:1000), CD81 (B-11) (Santa Cruz Biotechnology, sc166029, 1:20,000), His-tag (Cell Signaling, 2365S, 1:1000), HSP90 (C45G5) (Cell Signaling, 4877S, 1:1000), Syntenin (EPR8102) (Abcam, ab133267, 1:20,000), Rabbit IgG HRP-Conjugated (Abcam, ab16284, 1:2000), Rabbit IgG HRP-Conjugated (Cell signaling, 7074S, 1:5000), Mouse IgG HRP-Conjugated (R&D Systems, HAF007, 1:5000), ACE2 (R&D Systems, AF933, 1:1000), CD81 (GeneTex, GTX101766, 1:1000), CD63 (Proteintech, 25682-1-AP, 1:1000), GRP94 (1H10B7) (Proteintech, 60012-1-Ig, 1:1000), TSG101 (Proteintech, 14497-1-AP, 1:1000), Goat IgG HRP-conjugated (Thermo Fisher Scientific, 31410, 1:2000), Rabbit IgG HRP-Conjugated (Promega, W401B, 1:10,000) and Mouse IgG HRP-Conjugated (Promega, W402B, 1:10,000).<br>For flow cytometry: AF-647 mouse anti-human ACE2 (Clone # 535919)(R&D systems, FAB9332R, 0.4 µg/1M cells), AF-488 mouse anti-human ACE2 (Clone # 171607) (R&D systems, FAB9333G, 0.4 µg/1M cells), AF-647 isotype control mouse IgG2b (Clone # 20102) (R&D systems, IC003R, 0.4 µg/1M cells), AF-488 isotype control mouse IgG2b (Clone # 20102) (R&D systems, IC003G, 0.4 µg/1M cells).<br>For Micro Flow Vesiclometry (MFV) Analysis of EVs: AF-488 mouse anti-human ACE2 (Clone # 171607) (R&D systems, FAB9333G, 0.4 µg/2µg EVs as determined by nanodrop), APC mouse anti-human CD81 (Clone JS-81 (RUO)) (BD Biosciences, 561958, 1µL/2µg EVs as determined by nanodrop), AF-647 mouse anti-human CD63 (Clone H5C6 (RUO)) (BD, Biosciences, 561983, 2µL/2µg EVs as determined by nanodrop), AF-488 isotype control mouse IgG2b (Clone # 20102) (R&D systems, IC003G, 0.4 µg/2µg EVs as determined by nanodrop), APC isotype control mouse IgG1κ (Clone MOPC-21 (RUO)) (BD Biosciences, 555751, 1µL/2µg EVs as determined by nanodrop) and AF-647 isotype control mouse IgG1κ (Clone MOPC-21 (RUO)) (BD, Biosciences, 557714, 2µL/2µg EVs as determined by nanodrop).
Validation	These antibodies are provided by commercial vendors. Anti-ACE2 antibodies were validated by cells and EVs derived from cells over-expressing ACE2 (HEK-ACE2, HeLa-ACE2 and A549-hACE2). Anti-CD81, anti-CD63 and anti-Syntenin antibodies were validated on cell-derived EVs and small EV fractions. Anti-HSP90 antibody was validated by expression in non-vesicular fractions of cell-derived EVs. Anti-GRP-94 antibody was validated by expression on cells but not EVs.<br>ACE2 (Cell signaling, 4355S), CD81 (B-11) (Santa Cruz Biotechnology, sc166029), His-tag (Cell Signaling, 2365S), HSP90 (C45G5) (Cell Signaling, 4877S), Syntenin (EPR8102) (Abcam, ab133267), ACE2 (R&D Systems, AF933), CD81 (GeneTex, GTX101766), CD63 (Proteintech, 25682-1-AP), GRP94 (1H10B7) (Proteintech, 60012-1-Ig) and TSG101 (Proteintech, 14497-1-AP) were validated by the vendors by WB analysis of extracts of multiple cells lines.<br>AF-647 mouse anti-human ACE2 (Clone # 535919)(R&D systems, FAB9332R), AF-488 mouse anti-human ACE2 (Clone # 171607) (R&D systems, FAB9333G), APC mouse anti-human CD81 (Clone JS-81 (RUO)) (BD Biosciences, 561958), AF-647 mouse anti-human CD63 (Clone H5C6 (RUO)) (BD, Biosciences, 561983) were validated by flow cytometry in cell lines.

Eukaryotic cell lines

Policy information about cell lines

Cell line source(s)	The parent ACE2- human embryonic kidney HEK-293 cells (HEK) (ATCC, CRL-1573) or human cervical cancer HeLa cells (HeLa) (ATCC, CRM-CCL-2) are transduced with lentiviral pDual-ACE2 expression vector for stable ACE2 expression and production of ACE2+ EVs. Dr. Daniel Batlle and Dr. Jan Wysocki generously provide the HEK-293 cells overexpressing ACE2 (HEK-ACE2). Dr. Thomas Gallagher of Stritch Medical School, Loyola University kindly provided HeLa and HeLa-ACE2 cells via the Hope group. The wild-type SARS-CoV-2 live virus study was conducted at the NIAID-supported BSL-3 facility at University of Chicago Howard T. Ricketts Regional Biocontainment Laboratory using Vero-6 cells (ATCC, CRL-1586) and A549 cells (ATCC, CCL-185) overexpressing ACE2 (A549-hACE2) cells which were a kind gift of Benjamin TenOever, Mt Sinai Icahn School of Medicine.
Authentication	We have done western blotting and flow cytometry analysis for HEK, HEK-ACE2, HeLa and HeLa-ACE2. VERO-6 and A549-hACE2 cells were validated at University of Chicago Howard T. Ricketts Regional Biocontainment Laboratory for appropriate gene expression patterns by Ace2 Western Blotting and RNAseq analysis.
Mycoplasma contamination	No mycoplasma contamination as measured routinely in the laboratory.
Commonly misidentified lines<br>(See ICLAC register)	None.

Animals and other organisms

Policy information about studies involving animals; ARRIVE guidelines recommended for reporting animal research

Laboratory animals	For SARS-CoV-2 infection experiment: 6-9 weeks old female and male B6.Cg-Tg(K18-ACE2)2Prlmn/J (K18-hACE2) mice (Jackson Laboratory). All mice used in this study were housed in specific pathogen-free facilities, with regular diet and kept in light from 6am to 6pm at room temperature (around 22C) and humidity 41-42% in the Animal Resources Facilities at BSL-3 facility at University of Chicago Howard T. Ricketts Regional Biocontainment Laboratory.<br>For the biodistribution experiment: B6 mice (Jackson laboratory) (10 weeks old males) were used in the study. Animals were kept in specific pathogen-free facilities with regular diet and regular light/dark cycles, and regular ambient temperature and humidity in the Animal Resources Center at Northwestern University.
Wild animals	None.
Field-collected samples	None.
Ethics oversight	For SARS-CoV-2 infection experiment: All animal procedures conformed to the NIH Guidelines for the Care and Use of Laboratory Animals and were accepted by the University of Chicago Institutional Animal Care and Use Committees.

For the biodistribution experiment: All animal procedures were complied with the NIH Guidelines for the Care and Use of Laboratory Animals and were approved by the respective Institutional Animal Care and Use Committees.

Note that full information on the approval of the study protocol must also be provided in the manuscript.

Human research participants

Policy information about studies involving human research participants

Population characteristics	PreCOVID-19 patients (100% males, mean age 74 years old), COVID-19 convalescent (36.9% males, mean age 42.9 years old), and acute COVID-19 (56.5% males, mean age 61.5 years) patients and sero-negative donors (0% males, mean age 39.7 years).
Recruitment	For collecting human blood specimens, patients and donors were recruited at Northwestern Memorial Hospital based on their availability and willingness to consent and participate in the research. Participants were not compensated.
Ethics oversight	All research activities with human blood specimens of pre-COVID-19, sero-negative (healthy) donors, and acute and convalescent COVID-19 patients were implemented under NIH guidelines for human subject studies and the protocols approved by the Northwestern University Institutional Review Board (STU00205299 and #STU00212371) as well as the Institutional Biosafety Committee.

Note that full information on the approval of the study protocol must also be provided in the manuscript.

Clinical data

Policy information about clinical studies

All manuscripts should comply with the ICMJEguidelines for publication of clinical research and a completedCONSORT checklist must be included with all submissions.

Clinical trial registration	not applicable
Study protocol	Northwestern University IRB STU00205299, STU00212371, and STU00205299-MOD0001.
Data collection	All research activities with human blood specimens of pre-COVID-19, sero-negative (healthy) donors, and acute and convalescent COVID-19 patients were implemented under NIH guidelines for human subject studies and the protocols approved by the Northwestern University Institutional Review Board (STU00205299, STU00212371, and STU00205299-MOD0001) as well as the Institutional Biosafety Committee. For collecting human blood specimens, patients and donors were recruited at Northwestern Memorial Hospital based on their availability and willingness to consent and participate in the research. Participants were not compensated.
Outcomes	not applicable.

Flow Cytometry

Plots

Confirm that:

- [x] The axis labels state the marker and fluorochrome used (e.g. CD4-FITC).
- [x] The axis scales are clearly visible. Include numbers along axes only for bottom left plot of group (a 'group' is an analysis of identical markers).
- [x] All plots are contour plots with outliers or pseudocolor plots.
- [x] A numerical value for number of cells or percentage (with statistics) is provided.

Methodology

Sample preparation	Cells were blocked with mouse serum IgG (Sigma, 15381) for 10 min at room temperature and then incubated with specific antibodies; for 45 min on ice, followed by washing twice with 2% EV-free FBS/PBS. Cells were then diluted in 2% EV-free FBS/PBS, and DAPI was added as to exclude dead cells. Finally cells were filtered and analyzed using flow cytometry. Viable singlets were gated for percentage and mean fluorescence intensity (MFI) measurements ACE2+ cells.<br>For cell-based RBD binding neutralization by EVs and human plasma, the RBD-biotin-AF647 bait was incubated with EVs (ACE2+ and ACE2-), recombinant human ACE2 (rhACE2), or human plasma (10 µL or 80 µL) for 45 minutes on ice (creating “neutralized RBD”), then incubated with ACE2+ HEK-293 cells (200,000 cells in 100 µL 2% EV-free FBS/PBS) for 45 minutes on ice. RBD bait that was incubated with PBS, or with ACE2- EVs, non-fluorescent RBD bait (mock control) and ACE2- cells were used as controls. Cells were then spun and washed twice with 2% EV-free FBS/PBS or PBS. DAPI was added as to exclude dead cells analyzed on flow cytometer and viable singlets were gated for percentage and mean fluorescence intensity (MFI) measurements of the RBD-AF647+ population.
Instrument	BD FACSaria SORP and LSR-II.
Software	BD FACSDiva softwares v8.0.2 or v8.0.3 or Flow Jo v10.6.2

Cell population abundance	- ACE2+ cells in HEK-ACE2 and HeLa-ACE2 cells, 97 and 57-70% respectively.<br>- RBD bound cells in cell-based neutralization assays.
Gating strategy	DAPI negative viable single cells based on FSC, SSC and FSH are gated for percentage and mean fluorescence intensity (MFI).

☒ Tick this box to confirm that a figure exemplifying the gating strategy is provided in the Supplementary Information.
